# The Social Prescription: Enhancing Cognitive Functioning Through Social Activities

**DOI:** 10.1093/geroni/igaf122.562

**Published:** 2025-12-31

**Authors:** Meng Huo, Dawn Carr

## Abstract

Social activities have been linked to a variety of physical and emotional benefits in later life, but the contribution these activities can make to older adults’ cognitive health remains less clear. This symposium extends the growing body of work on social activities and cognitive functioning by showcasing four studies that considered the risk of Alzheimer’s disease and related dementias (ADRD). Brown examined older African Americans free of dementia at baseline, observing that their social activities led to slower cognitive declines over 10 years, regardless of the APOE genotype that independently affected cognitive changes. Han identified a robust within-person association between prosocial behaviors and slower cognitive declines, but this association was stronger among older adults at higher genetic risk for AD. Turning to older adults with mild cognitive impairment (MCI)—a common precursor of ADRD, Huo found that those who volunteered, particularly those who continuously volunteered or initiated volunteering, exhibited more positive cognitive changes. Amano further revealed the stability of older adults’ engagement in social activities in the presence of MCI, highlighting the importance of providing additional resources to those with limited social engagement. These studies reveal the crucial role social activities play in slowing cognitive declines in healthy adults and those already experiencing early declines that precede pathological deterioration. Findings identify social factors underlying risk and resilience as older adults experience cognitive changes and inform tailored, targeted interventions. Our discussant, Dr. Dawn Carr, will synthesize these studies, discuss their theoretical and practical applications, and address challenges and opportunities in future research.

